# A study of the composition of the Obsoletus complex and genetic diversity of *Culicoides obsoletus* populations in Spain

**DOI:** 10.1186/s13071-021-04841-z

**Published:** 2021-07-03

**Authors:** Cecilia Aguilar-Vega, Belén Rivera, Javier Lucientes, Isabel Gutiérrez-Boada, José Manuel Sánchez-Vizcaíno

**Affiliations:** 1 Animal Health Department, Faculty of Veterinary Medicine, VISAVET Health Surveillance Centre, Complutense University of Madrid, Madrid, Spain; 2 Department of Animal Pathology (Animal Health), Faculty of Veterinary Medicine, AgriFood Institute of Aragón IA2, University of Zaragoza, Zaragoza, Spain

**Keywords:** Obsoletus complex, Cytochrome *c* oxidase I gene, *Culicoides obsoletus*, *Culicoides scoticus*, *Culicoides montanus*, Bluetongue virus, Single-tube multiplex polymerase chain reaction

## Abstract

**Background:**

The *Culicoides obsoletus* species complex (henceforth ‘Obsoletus complex’) is implicated in the transmission of several arboviruses that can cause severe disease in livestock, such as bluetongue, African horse sickness, epizootic hemorrhagic disease and Schmallenberg disease. Thus, this study aimed to increase our knowledge of the composition and genetic diversity of the Obsoletus complex by partial sequencing of the cytochrome *c* oxidase I (*cox1*) gene in poorly studied areas of Spain.

**Methods:**

A study of *C. obsoletus* populations was carried out using a single-tube multiplex polymerase chain reaction (PCR) assay that was designed to differentiate the Obsoletus complex sibling species *Culicoides obsoletus* and *Culicoides scoticus*, based on the partial amplification of the* cox1* gene, as well as* cox1* georeferenced sequences from Spain available at GenBank. We sampled 117 insects of the Obsoletus complex from six locations and used a total of 238 sequences of *C. obsoletus* (*ss*) individuals (sampled here, and from GenBank) from 14 sites in mainland Spain, the Balearic Islands and the Canary Islands for genetic diversity and phylogenetic analyses.

**Results:**

We identified 90 *C. obsoletus *(*ss*), 19 *Culicoides scoticus* and five *Culicoides montanus* midges from the six collection sites sampled, and found that the genetic diversity of *C. obsoletus* (*ss*) were higher in mainland Spain than in the Canary Islands. The multiplex PCR had limitations in terms of specificity, and no cryptic species within the Obsoletus complex were identified.

**Conclusions:**

Within the Obsoletus complex, *C. obsoletus* (*ss*) was the predominant species in the analyzed sites of mainland Spain. Information about the species composition of the Obsoletus complex could be of relevance for future epidemiological studies when specific aspects of the vector competence and capacity of each species have been identified. Our results indicate that the intraspecific divergence is higher in *C. obsoletus* (*ss*) northern populations, and demonstrate the isolation of *C. obsoletus* (*ss*) populations of the Canary Islands.

**Graphical abstract:**

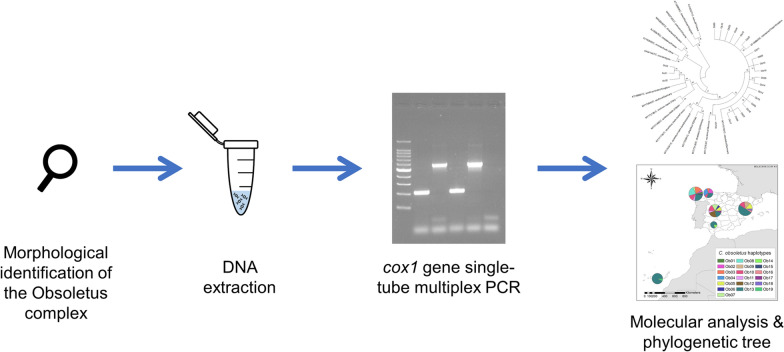

**Supplementary Information:**

The online version contains supplementary material available at 10.1186/s13071-021-04841-z.

## Background

Although some species of the genus *Culicoides* (Diptera: Ceratopogonidae) have been confirmed as biological vectors of numerous arboviruses of veterinary importance, the number of vector species is a very small proportion of the diversity of *Culicoides* species [1, 2]. Three of the arboviruses of the genus *Orbivirus* (family* Reoviridae*)—bluetongue virus (BTV), African horse sickness virus (AHSV) and epizootic hemorrhagic disease virus (EHDV) [3]—can have an impact on livestock welfare and cause significant economic losses, and are therefore listed as notifiable diseases by the World Organization for Animal Health [4]. BTV and EHDV affect mainly ruminants, with severe clinical disease observed in sheep and deer, respectively, whilst AHSV infects equids and can cause significant mortality in horses. BTV has been reported in every habitable continent, and since 1998, numerous BTV serotypes have been circulating in Europe [5]. Outbreaks of five BTV serotypes have been reported in Spain since 1956: BTV-10 (1956), BTV-2 (2000), BTV-4 (2003–2005; 2010–2020), BTV-1 (2007–2017; 2020) and BTV-8 (2008–2010; 2020) [6]. AHSV is endemic in sub-Saharan Africa, but outbreaks have occurred in northern African countries, the Middle East, southwest Asia, and southern regions of Europe [7]. In Spain, two epizootics, with different serotypes, occurred in 1966 (AHSV-9) and 1987–1989 (AHSV-4) [7]. EHDV has been reported in North and South America, Australia, Africa and Asia [8]. Despite not being reported in the European Union, EHDV outbreaks have been declared in Mediterranean Basin countries [9]. This proximity suggests a risk of introduction of EHDV into the EU, as has occurred with AHSV [10] and BTV [10, 11].

*Culicoides imicola*, a member of the subgenus *Avaritia*, is deemed the major BTV, AHSV and EHDV vector in Africa, the Middle East, southeast Asia and southern Europe [5, 12]. *C. imicola* is highly abundant in central and southern regions of mainland Spain [13, 14]. Carpenter et al. [15] showed the vector competence of several Palearctic species of *Culicoides* prior to the introduction of BTV-8 into northern Europe. After the BTV-8 epizootic in northern Europe, several species of different subgenera were implicated as potential competent BTV vectors in the field: *Culicoides chiopterus* [16],* Culicoides dewulfi*, and the *Culicoides obsoletus* species complex (henceforth ‘Obsoletus complex’) of the subgenus *Avaritia* [17]; and the *Culicoides pulicaris* species complex (henceforth ‘Pulicaris complex’) [17] of the subgenus *Culicoides*. In areas of northern European, the Obsoletus complex is considered of great importance in BTV transmission due to its predominance and prevalence in entomological surveys [18], and its vector competence [15, 19, 20]. The Obsoletus complex is widespread in Spain, although it is most abundant in northern regions of the country [13, 14]. Moreover, *C. obsoletus* breeds in diverse habitats with high organic content, including livestock manure [21–23], and it can breed in indoor locations [24], which implies a great risk of BTV transmission to livestock.

Females of the species that belong to the Obsoletus complex (*C. obsoletus* and *C. scoticus* and cryptic species/clades within the complex) are difficult to distinguish morphologically [25, 26]. Thus, many studies aimed at assessing oral susceptibility to BTV and EHDV in these sibling species have been performed at the species complex level [15, 20, 27]. Other studies have studied vector competence at species level within the complex, but with a low number of insects [19, 28]. Those studies proved the susceptibility of the Obsoletus complex to different BTV serotypes [15, 19, 20, 28], and one EHDV serotype [27]. To our knowledge, no study has been carried out on the oral susceptibility of the Obsoletus complex to AHSV, but one study achieved the isolation of AHSV from pools consisting mainly of the Obsoletus and Pulicaris complexes during the 1987–1989 Spanish epizootic [29]. However, sibling species within the complex may have different vector competence and capacities [30].

The mitochondrial DNA (mtDNA) cytochrome *c* oxidase I (*cox1*) gene is widely used for the molecular identification of species and to study their genetic diversity [31], and is also frequently used to study the diversity of the Obsoletus complex [2, 32–37]. Few studies have yielded Obsoletus complex sequences of * cox1* in Spain [32–34], although a recent study investigated the genetic diversity of the Obsoletus complex at a large scale using insects from 20 different countries, including Spain [35]. The majority of available Spanish* cox1* Obsoletus complex sequences have come from Catalonia and the Balearic Islands [33, 35], southeastern areas of Spain [35] and the Canary Islands [32]. The importance of the Obsoletus complex as a biological vector of pathogens that cause severe diseases in livestock [1] justifies the aim of this study to expand our knowledge of the composition and genetic diversity of the Obsoletus complex by using the partial sequencing of the* cox1* gene in poorly studied areas of Spain, as well as to compare our results with georeferenced sequences from Spain that have been deposited in GenBank [38]. Moreover, we developed a multiplex polymerase chain reaction (PCR) to facilitate the differentiation of *C. obsoletus* from *C. scoticus* in the region.

## Methods

### Specimen collection and identification

**Fig. 1:**
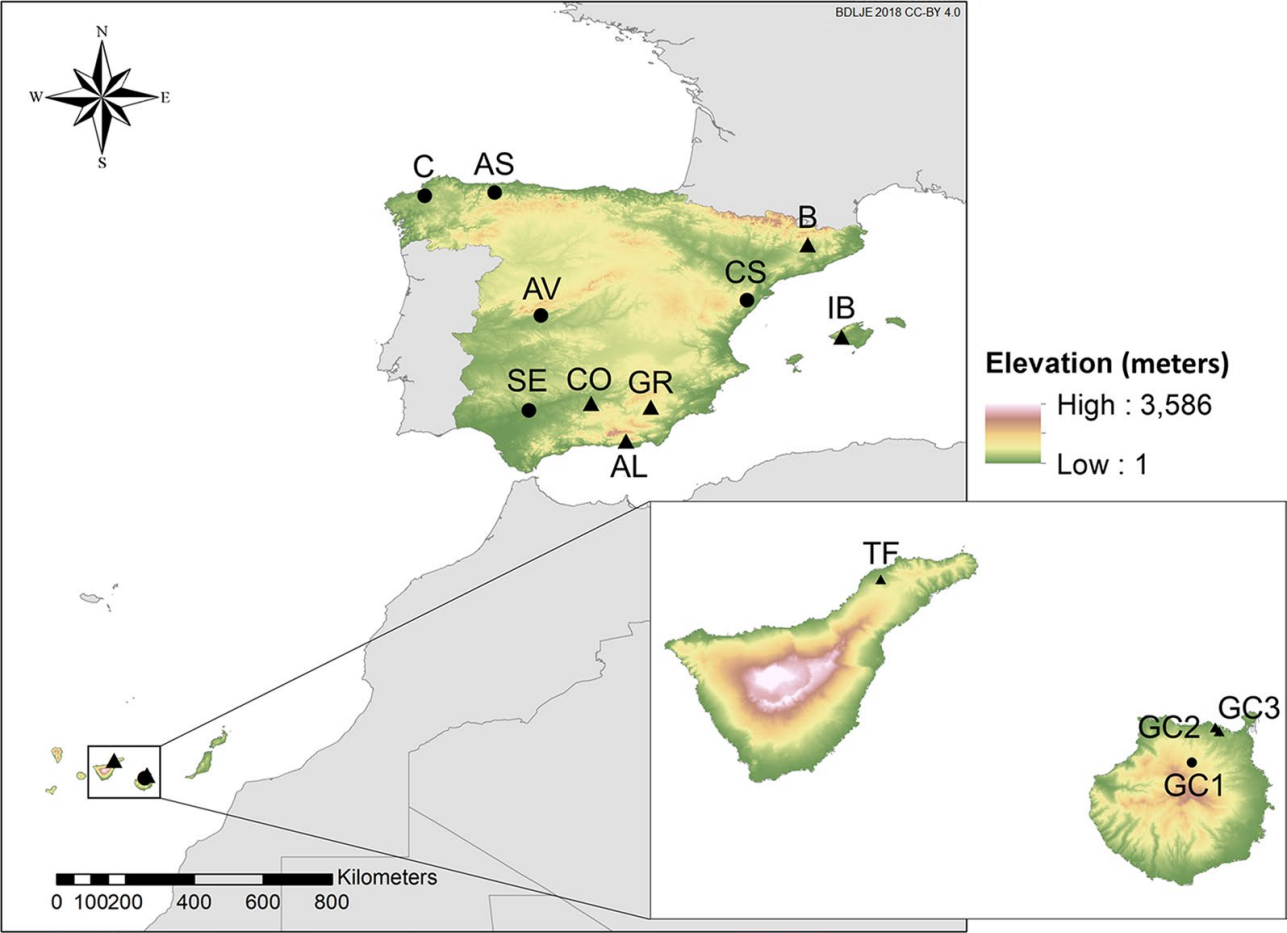
Location of the collection sites of the midges of the Obsoletus complex used in this study (*black dots*) and of the georeferenced sequences retrieved from GenBank (*black triangles*). Elevation of the study area is shown at 15-arc second resolution (GMTED2010 image courtesy of the US Geological Survey [42]). Locations are coded based on the province’s location (see Table 1): Barcelona (*B*), Mallorca (*IB*), Córdoba (*CO*), Granada (*GR*), Almería (*AL*), Tenerife (*TF*), Gran Canaria (*GC2*,* GC3*). Spanish administrative boundaries were provided by the Instituto Geográfico Nacional (ign.es) (BDDAE CC-BY 4.0)

The 117 insects that were analyzed in the current study originate from mainland Spain (they were provided by the Spanish Bluetongue National Surveillance Program), and from a trap that we placed in the Canary Islands (Table 1). Figure 1 shows the location of the collection sites used in this study and those of the georeferenced sequences retrieved from GenBank (Additional file 1: Table S1) [38]. United States Centers for Disease Control and Prevention ultraviolet light traps were located in all six collection sites at 1.7- to 2-m height from dusk until dawn close to animal holdings with more than ten susceptible hosts [39]. Insects were preserved in 70% ethanol prior to identification, and we analyzed females identified as belonging to the Obsoletus complex using different morphological identification keys [40, 41].

**Table 1:** Location of the collection sites of the tested midges of the *Culicoides obsoletus* species complex (Obsoletus complex)

ID	Province	Municipality	Coordinates^a^	Date of collection	No. of midges of the Obsoletus complex	No. of *Culicoides obsoletus*	No. of *Culicoides scoticus*	No. of *Culicoides montanus*	No. of unidentified *Culicoides*
CS	Castellón	Xert	40.53º, 0.13º	20 August 2009	25	22	1	2	0
AS	Asturias	Tineo	43.34º, − 6.48º	13 July 2017	14	10	4	0	0
AV	Ávila	Candeleda	40.18º, − 5.28º	16 August 2017	18	18	0	0	0
C	La Coruña	Mabegondo	43.21º, − 8.29º	6 September 2009	26	22	1	3	0
SE	Seville	Lora del Río	37.67º, − 5.51º	22 May 2007	8	5	0	0	3
GC1	Las Palmas	Valleseco	28.04º, − 15.58º	26 September 2018	26	13	13	0	0

For the geographical location of the study sites, see Fig. 1

*ID* Identifier

^a^Coordinates are in decimal degrees and correspond to the centroid of the municipality

### DNA extraction and molecular identification

DNA extraction was performed using the High Pure PCR Template Preparation Kit (Roche Diagnostics, Mannheim, Germany) following the manufacturer’s instructions. Specimens had previously been removed from ethanol, dried on absorbent paper, and then homogenized in 100 μl of binding buffer.

**Fig. 2:**
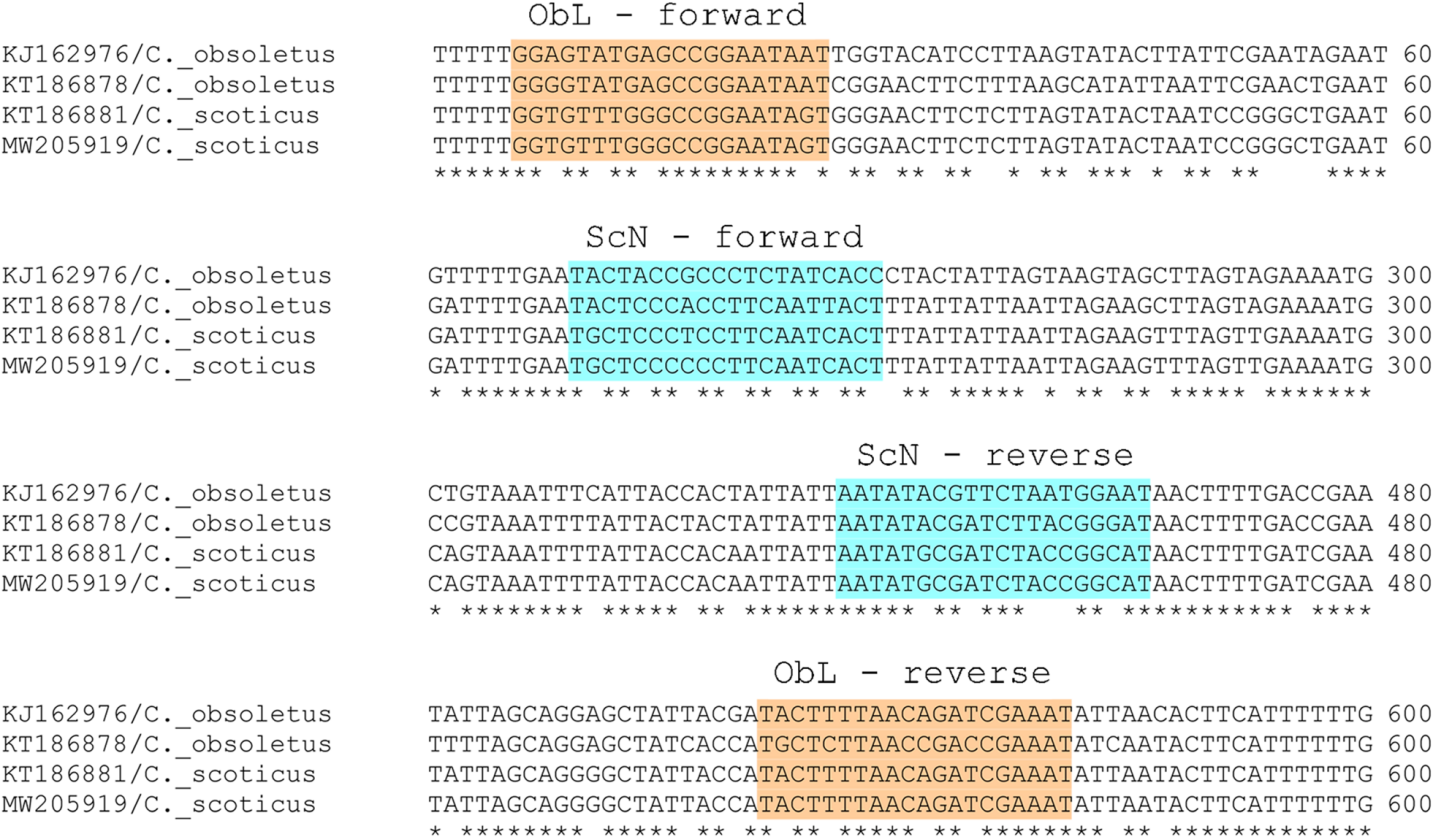
*Culicoides obsoletus* (*ObL*) and *Culicoides scoticus* (*ScN*) primer location in aligned *C. obsoletus* and *C. scoticus* sequences. Only the part of the alignment corresponding with the primer location is shown. Alignment was performed using Clustal Omega software (https://www.ebi.ac.uk/Tools/msa/clustalo/)

For the partial amplification of the mtDNA* cox1* region, we designed two pairs of primers using Primer3Plus v.2.4.2. software [43]. For *C. obsoletus* (ObL), forward (5′-GGRGTATGAGCCGGAATAAT-3′) and reverse (5′-ATTTCGRTCDGTTAARAGYA-3′) primers were designed with a product size of ~ 576 base pairs (bp). For *C. scoticus* (ScN), we designed forward (5′-TGCTCCCYCCTTCAATCACT-3′) and reverse (5′-ATGCCGGTAGATCGCATATT-3′) primers to amplify a shorter sequence (~ 217 bp) of the* cox1* region. We checked the sensitivity and specificity of the primer pairs from European *C. obsoletus* and *C. scoticus* sequences available in GenBank [38] (https://www.ncbi.nlm.nih.gov/genbank/) (Fig. 2). PCR amplification was performed in a 25-μl final volume using the following reagents per reaction: 2 μl of Nuclease-free water, 15 μl of Platinum Green Hot Start PCR 2X Master Mix (Invitrogen, Lithuania), 1.5 μl of 20 μM forward primers (ObL and ScN), 1.5 μl of 20 μM reverse primers and 2 μl of genomic DNA. The amplification program was as follows: an initial denaturation step at 94 ºC for 5 min, 40 cycles of denaturation at 94 ºC for 1 min, annealing for 1 min and extension at 72 ºC for 1 min, and a final extension step at 72 ºC for 7 min. We optimized the annealing temperature to enhance the specificity to evaluate different annealing temperatures from 54 to 60 ºC in increments of 2 ºC. PCR products were stored at 4 ºC until the amplification of the PCR products was confirmed using electrophoresis on 2% agarose 1x TAE gel that contained SYBR Safe DNA Gel Stain (Invitrogen, USA), with a 100-bp DNA molecular weight marker (Takara). PCR products were purified using QIAquick PCR Purification kit (Qiagen, Germany) in the absence of primer dimer formation. Alternatively, gel bands were purified using QIAquick Gel Extraction kit (Qiagen) and forward strands were externally sequenced by Sanger sequencing using the ObL forward primer. We assigned the *Culicoides* species level using BLASTN+ 2.10.1 nucleotide [44].

### Molecular analysis and phylogenetic tree

For the molecular analyses and intraspecific sequence polymorphism of *C. obsoletus* (*ss*), we included* cox1* georeferenced sequences from Spain of *C. obsoletus*, which are available from the GenBank database (https://www.ncbi.nlm.nih.gov/genbank/). DNA polymorphism and haplotype diversity were obtained using DnaSP v.6.12. [45]. A median-joining network for the identified haplotypes was built using Network software v.10.2.0.0 [46], to show the relationships between them. Pairwise genetic differentiation was assessed using the fixation index (*F*_ST_) calculated in DnaSP [45]. *F*_ST_-values can range between 0 and 1, where 0 shows no differentiation, and 1 no genetic diversity shared between populations [47].

Some cryptic taxa in the Obsoletus complex have been previously identified [35, 48–50]: *C. obsoletus* O1 [*C. obsoletus *(*ss*); MT170705], *C. obsoletus* O2 (MT173130 and MT172091) and *C. obsoletus* O3 or clade dark (MT170541 and MT171736). We included representative sequences of each for the construction of our phylogenetic tree, as well as of *C. montanus* (MT172763), *C. scoticus* (MT170272) and the closely related *C. scoticus* clade 2 (MT171305 and MT172198) that is considered to be an intraspecific variant of *C. scoticus* [35]. We also included *Culicoides dewulfi* (KT186808 and KJ162977), *Culicoides chiopterus* (KJ162976 and MW205937), *Culicoides imicola* (KX641487 and KJ162982) and *Culicoides pulicaris* (MW207302) sequences.

All phylogenetic analyses were performed in MEGA X [51]. Sequences were aligned using the MUSCLE algorithm [52], and the suitability of the alignment was evaluated through the average evolutionary divergence for all nucleotide sequence pairs (*p*-distance). To generate a reliable phylogenetic tree, we set the threshold value for the *p*-distance at < 0.8 [53, 54]. We inferred a maximum likelihood (ML) phylogenetic tree with the general time reversible model [55] with a gamma distribution and invariant sites, and using 2000 bootstrap replications for reliability. Tree branches with bootstrap values inferior to 50% were collapsed to form a consensus tree [56].

## Results

### *cox1* multiplex PCR optimization

**Fig. 3:**
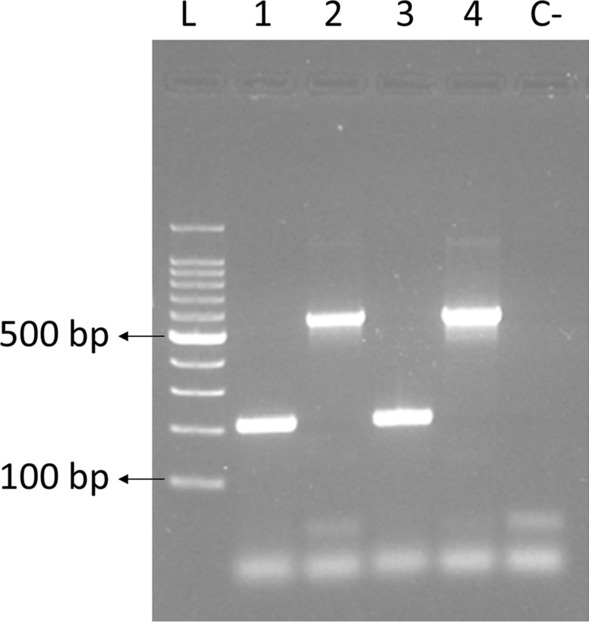
Validation of the optimized cytochrome *c* oxidase I (*cox1*) multiplex polymerase chain reaction assay with an annealing temperature of 60 ºC. *Lane L* 100-base pair (bp) ladder, *lanes 1* and* 3* *C. scoticus* individuals from GC1, *lanes 2* and* 4* *C. obsoletus* individuals from GC1, *lane C-* negative control of the PCR. The multiplex PCR included forward and reverse ObL and ScN primers. For other abbreviations, see Figs. 1 and  2

We evaluated four annealing temperatures in our multiplex* cox1* PCR: 54, 56, 58 and 60 ºC. The annealing temperature which improved the specificity was 60 ºC (Fig. 3). For every other temperature, non-specific PCR bands were obtained for *C. scoticus* samples. Hence, the optimal annealing temperature for maximal specificity was 60 ºC. However, for poor-quality samples, we found that it might be advisable to decrease the annealing temperature in order to increase sensitivity.

For *C. scoticus* from the Canary Islands site, ObL primers amplified a ~ 576-bp band with an annealing temperature of 54 ºC. These bands were purified and sequenced using the ObL forward primer. Thus, sequences of *C. scoticus* from that location were obtained and included in further analyses. The amplification of some *C. scoticus* sequences with an annealing temperature of 54 ºC made it necessary to increase the temperature to 60 ºC to be able to correctly differentiate the species and prevent non-specific PCR bands.

### Obsoletus complex sequence diversity for sampled sites

Of the 117 Obsoletus complex individuals studied here, 90 were identified as *C. obsoletus* (76.92%), 19 as *C. scoticus* (16.24%) and five as *C. montanus* (4.27%); three could not be identified (2.56%). The proportion of each species differs greatly in the six sites chosen for the study (Table 1). In SE and AV, all identified insects were *C. obsoletus*, while in GC1 half of the individuals were *C. scoticus*. In AS, CS and C, we identified a total of six *C. scoticus* individuals. *C. montanus* was identified in CS and C.

**Fig. 4:**
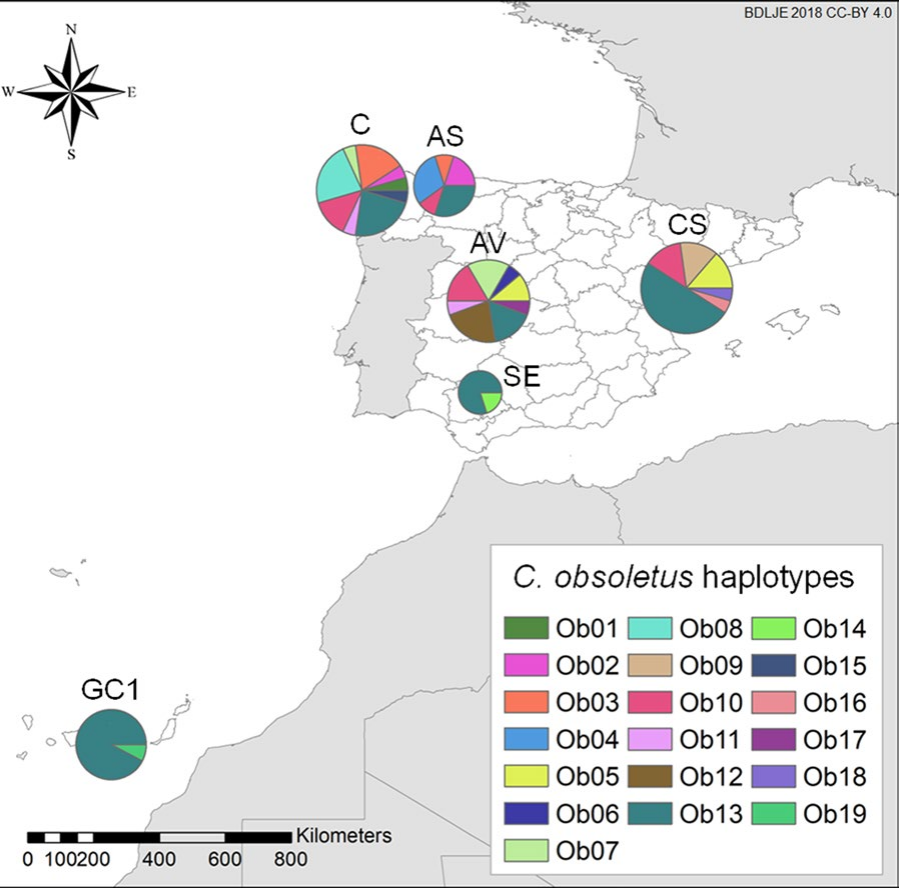
Distribution of the 19 different *C. obsoletus* haplotypes identified in this study. Differences in size of the pie charts represent the number of individuals sampled per site. Spanish administrative boundaries were provided by the Instituto Geográfico Nacional (ign.es) (BDDAE CC-BY 4.0). For abbreviations, see Fig. 1

We obtained a total of 90 sequences for *C. obsoletus*, 13 for *C. scoticus* of the Canary Island location, and five for *C. montanus*. All 108 sequences had a length  of 514 bp. We found 19 haplotypes for *C. obsoletus* (Ob01-Ob19), three for *C. scoticus* (Sc01-Sc03) and one for *C. montanus*. Sequences from each haplotype were deposited in GenBank under the following accession numbers: *C. obsoletus* (MW602810-MW602828), *C. scoticus* (MW602829-MW602831), and *C. montanus* (MW602832). The most prevalent *C. obsoletus* haplotype was Ob13, which was present at all six sampling sites (Fig. 4), and represented 42.22% (38/90) of all *C. obsoletus* sequences. The Ob13 haplotype was predominant at GC1 (12/13–92.31%), SE (4/5–80%) and CS (11/22–42.22%). Ob10 was found at the four northern locations, while Ob02 and Ob03 were found in C and AS, Ob05 in AV and CS, and Ob07 and Ob11 in AV and C. Ob08 was only found at C, Ob012 at AV, and Ob04 at AS, but at a relatively high proportion: 5/22 (22.72%), 4/18 (22.22%) and 3/10 (30%), respectively, at each site. The rest of the haplotypes were only found at one site in low numbers. For the *C. scoticus* population in the GC1 population, 11/13 individuals belonged to haplotype Sc03; in contrast, Sc01 and Sc02 haplotypes were are only represented by one individual.

We found that the interspecific nucleotide divergence was greater than the intraspecific diversity, as previously demonstrated [2, 35]. The total nucleotide diversity for all of the sequences was 0.0307 ± 0.0053, while for the *C. obsoletus* (*ss*) sequences it was 0.0045 ± 0.0004, and for *C. scoticus* 0.0006 ± 0.0003. Table 2 shows the sequence polymorphism of *C. obsoletus* (*ss*) populations obtained in the present study and from georeferenced sequences retrieved from GenBank.

**Table 2:** Intraspecific polymorphisms of the sequences of *C. obsoletus* (*ss*) populations evaluated in this study and of georeferenced sequences retrieved from GenBank

ID^a^	Number of sequences	GC content	*h*	Hd (SD)	*S*	π (SD)
AL (Genbank)	6	0.339	2	0.600 (0.129)	2	0.00263 (0.00056)
AS (this study)	10	0.340	4	0.822 (0.072)	3	0.00297 (0.00049)
AV (this study)	18	0.338	8	0.895 (0.038)	7	0.00455 (0.00064)
B (Genbank)	30	0.339	11	0.851 (0.046)	10	0.00442 (0.00048)
C (this study)	22	0.339	8	0.823 (0.050)	10	0.00465 (0.00082)
CO (Genbank)	13	0.339	4	0.731 (0.079)	5	0.00424 (0.00072)
CS (this study)	22	0.339	6	0.723 (0.085)	8	0.00492 (0.00071)
GC1 (this study)	13	0.339	2	0.154 (0.126)	1	0.00034 (0.00028)
GC2 (Genbank)	37	0.339	2	0.054 (0.050)	1	0.00012 (0.00011)
GC3 (Genbank)	11	0.339	2	0.182 (0.144)	1	0.00040 (0.00031)
GR (Genbank)	16	0.339	4	0.617 (0.096)	5	0.00315 (0.00068)
IB (Genbank)	26	0.339	4	0.625 (0.054)	5	0.00324 (0.00051)
SE (this study)	5	0.339	2	0.400 (0.237)	3	0.00263 (0.00156)
TF (Genbank)	9	0.339	1	–	0	–
Total	238	0.339	23	0.690 (0.028)	22	0.00372 (0.00022)

The analysis was performed using a 457-bp alignment

*h* Number of haplotypes, *Hd* haplotype (gene) diversity, *S* number of variable sites, π nucleotide diversity (per site)

^a^Identifies the geographical location (see Fig. 1)

### *C. obsoletus* sequence diversity compared with external Spanish sequences

**Fig. 5:**
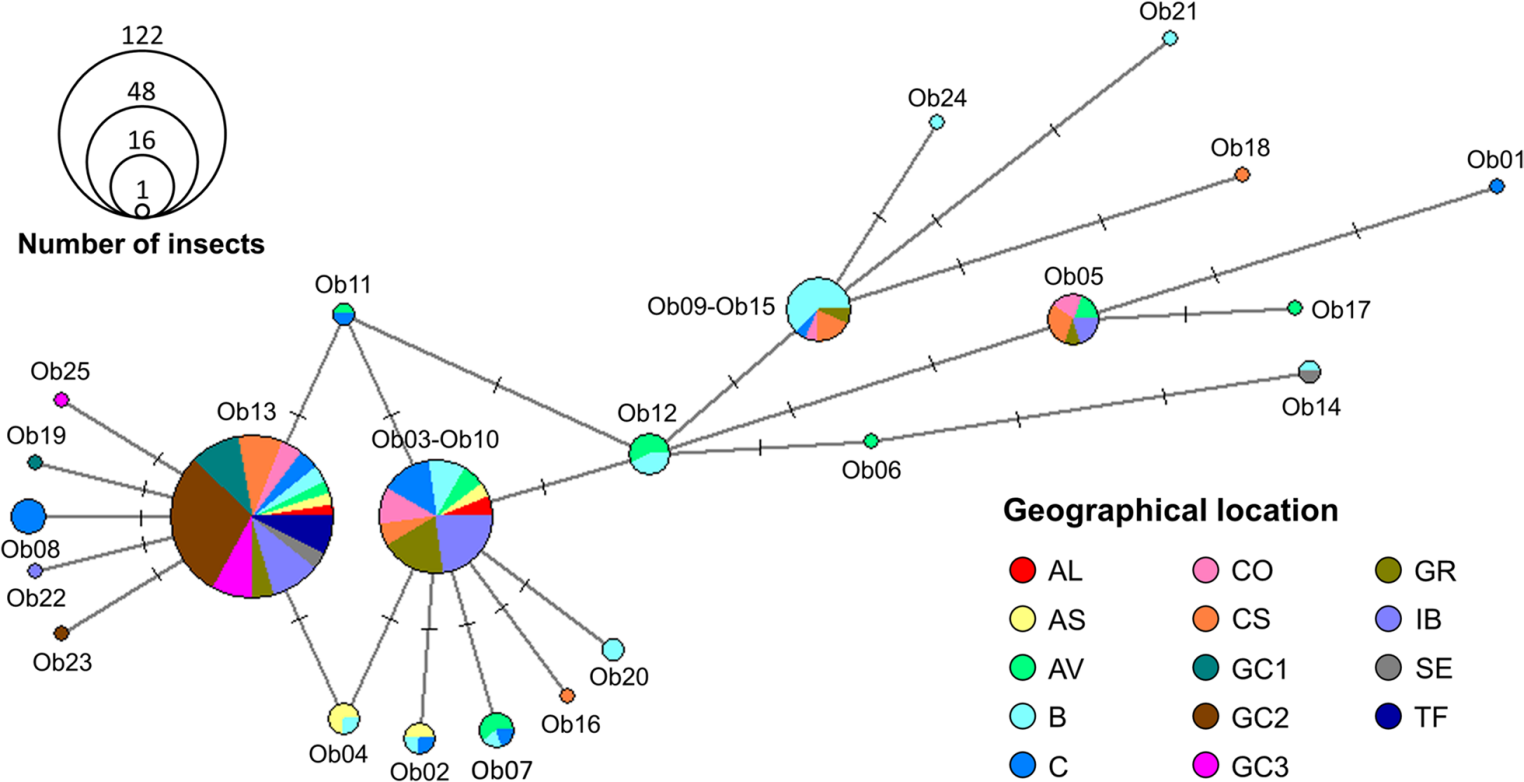
Median-joining network showing the relationship of the 23 haplotypes of 457-bp in length. Node size represents the number of sequences that comprise each haplotype. Each geographical location is color coded to show its haplotype frequencies. Perpendicular lines indicate nucleotide changes. For abbreviations, see Fig. 1

We obtained 148 *C. obsoletus* (*ss*) georeferenced sequences from Spain from GenBank (Additional file 1: Table S1), and aligned them with our sequences, which gave a total of 238 sequences. The final length of the alignment was 457 bp, and we identified 23 *C. obsoletus* (*ss*) haplotypes. Of 148 external sequences, 141 corresponded to one of the haplotypes identified in this study. We maintained the numbering of the identified haplotypes and added consecutive numbers for the new haplotypes identified from the GenBank sequences (Ob20–25). Due to the similarities of the sequences obtained in this study (Additional file 2: Table S2) and their shortening, three pairs of haplotypes had the same sequence in the 457-bp alignment: haplotypes Ob03 and Ob10 (Ob03–Ob10), Ob09 and Ob15 (Ob09–Ob15), as well as Sc01 and Sc03 (Sc01–Sc03). Figure 5 shows haplotype relationships determined using a median-joining network. Ob13 was the most frequent haplotype, comprising 51.26% (122/238) of the sequences, and was present at all collection sites. Ob03–Ob10, Ob09–Ob15 and Ob05 also occurred at a relatively high frequency, i.e. in ten or more insects. Eleven haplotypes were from only one individual: six of those identified in our samples and five from GenBank georeferenced sequences.

We found more *C. obsoletus* (*ss*) haplotypes and genetic diversity at B, C, AV, CO and CS (Table 2). However, SE, AL and the Canary Islands (GC1-3 and TF) populations had significantly lower intraspecific diversity, although few sequences were included for SE and AL. A pronounced level of genetic differentiation was observed between each Canary Island population, mainland Spain and IB populations in this study with *F*_ST_ values > 0.25, as well as between SE and AS, AV, B and GR (Table 3). Negative *F*_ST_ values can be a result of a small sample size in relation to a high number of haplotypes in the population, and should be considered as indicating no genetic differences [57]. Thus, no divergence was found among the Canary Island sites, or with SE, or between some populations on mainland Spain.

**Table 3:** Genetic differentiation based on pairwise *F*_ST_ values

ID^a^	AL	AS	AV	B	C	CO	CS	GC1	GC2	GC3	GR	IB	SE	TF
AL														
AS	− 0.065													
AV	0.106	0.174												
B	0.181	0.223	0.076											
C	− 0.045	0.033	0.083	0.145										
CO	− 0.045	0.045	− 0.004	0.08	− 0.008									
CS	0.015	0.106	0.059	0.091	0.025	− 0.031								
GC1	0.371	0.452	0.515	0.555	0.317	0.383	0.28							
GC2	0.389	0.465	0.526	0.566	0.325	0.394	0.289	0						
GC3	0.367	0.447	0.511	0.552	0.314	0.379	0.278	0	0					
GR	− 0.057	0.027	0.033	0.103	0.01	− 0.045	0.041	0.513	0.53	0.509				
IB	− 0.089	0.02	0.095	0.183	− 0.005	− 0.031	0.014	0.356	0.371	0.352	− 0.003			
SE	0.2	0.274	0.359	0.388	0.198	0.234	0.15	0	0	0	0.34	0.201		
TF	0.4	0.479	0.532	0.573	0.332	0.401	0.294	0	0	0	0.539	0.379	0	

^a^For geographical location, see Fig. 1

### Phylogenetic analysis

**Fig. 6:**
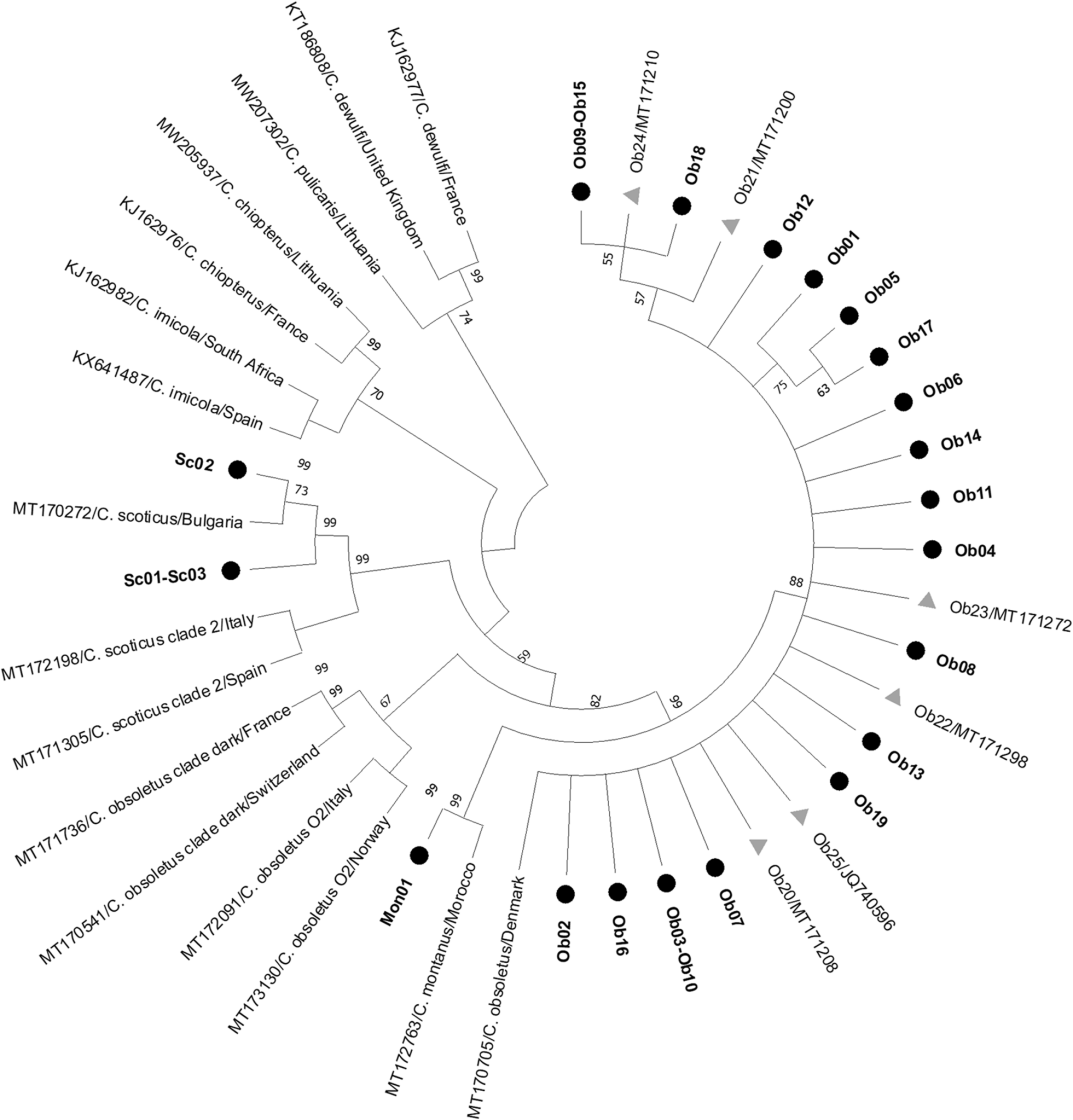
Inferred phylogenetic tree for the partial mitochondrial DNA* cox1* gene using the maximum likelihood method, the general time reversible model with a gamma distribution and invariant sites and 2000 bootstrap replications. Reference sequences retrieved from GenBank (https://www.ncbi.nlm.nih.gov/genbank/) include accession number, species and country information. Haplotypes identified in this study (Ob01–Ob19) are highlighted in* bold* and by a* black circle*, while Spanish haplotype sequences identified from retrieved GenBank sequences (Ob20–Ob25) are marked by a* gray triangle* and include the accession number

The alignment was suitable for the generation of reliable phylogenetic trees, with an average of 0.09 nucleotide substitutions per site when taking into consideration all sequence pairs. The ML phylogenetic tree (Fig. 6) showed that the sequences analyzed here have a small evolutionary distance; this was also supported by the low nucleotide diversity (Additional file 2: Table S2). The tree also corroborates the absence of cryptic species in the area of study.

## Discussion

In the present study, a single-tube multiplex PCR assay based on the partial amplification of the mtDNA* cox1* gene was developed. Previous studies have also developed single-tube multiplex PCR assays for the* cox1* gene for the Obsoletus complex [37, 58, 59]. However, the amplicon size for *C. obsoletus* used in these assays was inadequate for our posterior sequence diversity and phylogeographic analyses. Our PCR allows the differentiation of *C. obsoletus* from *C. scoticus* within the Obsoletus complex, without the need for sequencing derived from the use of generic primer pairs such as LCO/HCO [60], C1-J-1718/C1-N-2191 [61, 62], and Lep [63], although ObL primers showed a lack of specificity for the amplification of *C. montanus*, which we discuss below. We found some cross-reactivity with the ObL primers and *C. scoticus* samples at low annealing temperatures, although we designed the primers so as to avoid this. This cross-reactivity was overcome by using an annealing temperature of 60 ºC (Fig. 3). Other authors have pointed out similar difficulties concerning the specificity of specifically designed primers for *Culicoides* species [48]. We did not evaluate whether our primers could amplify other *Culicoides* species outside the Obsoletus complex, thus previous morphological identification cannot be substituted by our molecular approach. Moreover, morphological identification of all the midges as belonging to the Obsoletus complex coincided with their molecular identification. The correct morphological identification is key to accurately estimate the large-scale distribution of *Culicoides* species and complex when molecular identification is not achievable.

Primers designed for *C. obsoletus* (ObL) also amplified one haplotype of *C. montanus*. The existence of other cross-reactions cannot be ruled out for other Obsoletus complex species haplotypes that were not identified in this study for Spain, or for other geographical regions. To overcome the limitations regarding specificity that were encountered for *C. montanus* and some haplotypes of *C. scoticus* at low annealing temperatures of the applied primers, a multi-marker approach including other mitochondrial (*cox2* and* cytb*) and nuclear loci (ITS1 and ITS2) should be considered for future studies in which the main goal is to correctly differentiate between *C. obsoletus* (*ss*), C*. scoticus*, *C. montanus* and all cryptic species within *C. obsoletus* [64]. To our knowledge, no PCR for the* cox1* gene that has been designed enables discrimination of *C. montanus* within the Obsoletus complex/group [37, 48, 58, 59]. Due to the low genetic distance between *C. obsoletus* (*ss*) and *C. montanus* (Fig. 6; Additional file 2: Table S2) [35], it is highly unlikely that specifically generated primers created for *C. obsoletus* (*ss*) do not also amplify *C. montanus*, especially if the latter species has not been taken into account in the primer design, as our results show. Some authors have been able to correctly differentiate *C. montanus* from *C. obsoletus* using the ITS1 [67] and ITS2 markers [66, 68], although other studies faced similar specificity limitations to ours when identifying *C. montanus* in a PCR designed specifically for *C. obsoletus* that used the ITS2 marker [69].

The prevalence of *C. montanus* is generally low in entomological surveys, with the exception of some studies carried out in Morocco [35]. Therefore, its role as an arbovirus vector has not yet been defined. In addition, the taxonomic status of *C. montanus* in western Palearctic regions has been questioned by some authors due to the low genetic distance found between it and *C. obsoletus* (*ss*) when using different molecular markers [35, 65, 66].

We found higher genetic diversity in mainland Spain than in the Canary Islands (Table 2). The low genetic diversity, inexistent genetic differentiation among the structure of *C. obsoletus* (*ss*) in all Canary Islands populations, and strong divergence with the other sampling sites (Tables 2, 3) are probably a consequence of their isolation, which implies inbreeding and low gene flow outside the islands [70]. On the other hand, for site IB there was no to low differentiation with mainland Spain, except for site B with which there was moderate divergence, as it had three of the most prevalent haplotypes in this study: Ob03–Ob10, Ob05 and Ob13 (Fig. 5). Its closeness to mainland Spain may facilitate gene flow between midge populations of the two. Haplotype and nucleotide diversity in the south of mainland Spain is lower than in northern areas of Spain. These differences can be attributed to sample size; however, the abundance of the Obsoletus complex is higher in northern and mountainous areas of Spain [14], and genetic diversity is partially influenced by population size [70]. In addition to differences in population size, orographic barriers such as altitude (Fig. 1), might limit genetic flow between some of the mainland populations in this study, although the limited sample size of some collection sites prevents us from drawing a firm conclusion (Table 3).

We found similar overall proportions of each species to those reported in recently published work [35]. In Mignotte et al. [35], of the 179 sequences analyzed for samples from Spain, 128 (71.51%) were from *C. obsoletus*, two from (1.12%) *C. montanus*, 28 (15.64%) from *C. scoticus* and 21 (11.73%) from *C. scoticus* clade 2, although in a site in Catalonia, only *C. scoticus* midges were identified. Moreover, there are dissimilar ratios for the Canary Islands between our study and that of Mignotte et al. [35], since we identified a 1:1 ratio for *C. obsoletus* (*ss*) and *C. scoticus*, while in the latter study, *C. scoticus* was not found. The peculiar orographic characteristics of the Canary Islands justify strengthening our knowledge of the Obsoletus complex composition there, in collection sites that are more homogeneously distributed. We did not find *C. scoticus* clade 2, although we did not obtain a large sequence for any of the six *C. scoticus* midges from mainland Spain. Moreover, we did not find evidence of any cryptic species at any of the sampled sites, as in studies carried out in nearby countries, such as Morocco [35, 71]. Both *C. obsoletus* O2 and *C. obsoletus* clade dark or O3 have been described from Switzerland, Sweden, Denmark, Finland, Latvia, Norway, France and Italy at latitudes above 45º [35, 48, 50]. The former has also been described from Poland [35], and *C. obsoletus* clade dark or O3 from the Netherlands [49]. In the United Kingdom, evidence of Obsoletus complex cryptic taxa has also been found [36]. Cryptic species may have significant epidemiological differences in terms of vector competence, host preferences and breeding sites, although there is as yet no evidence for this [30]. Our results, along with those of previous work carried out on Spanish populations of the Obsoletus complex [33–35], show that *C. obsoletus* cryptic species O2 and clade dark might be absent from Spain, present in very low numbers, or present in unsampled locations. The potential absence of cryptic taxa from the Obsoletus complex in Spain may be of relevance to future epidemiological studies, as species-specific vector competence within the complex could be examined by using a greater number of insects [19, 28]. Nevertheless, more *C. obsoletus* sequences should also be acquired to definitively exclude the presence of cryptic species in areas with a high abundance of the Obsoletus complex, namely, northern locations [13, 14], which also show more genetic diversity (Table 2).

According to the entomological survey which has been conducted by the Spanish Bluetongue National Surveillance Program since 2004, the Obsoletus complex is the most abundant in northern areas of mainland Spain and the *C. imicola* complex is generally absent or found in low numbers [14]. Therefore, it is thought that the Obsoletus complex plays a key role in the transmission of BTV there, given that it is a competent vector of the virus [15, 19, 20]. The persistence of BTV in northern Spain was empirically proven by the circulation of BTV-1 from 2007 to 2009 and BTV-8 in 2008 and 2020 [72]. However, the exact contribution of the Obsoletus complex to BTV transmission in central and southern areas of mainland Spain, where *C. imicola* is the main vector of BTV, has not yet been well defined [13, 73].

## Conclusions

Our study reveals that *C. obsoletus* (*ss*) is the predominant species within the Obsoletus complex in mainland Spain. This information may be of relevance for future epidemiological studies when more robust information is available on specific aspects of the vector capacity (vector competence, adult longevity, biting rate [1]) of each of the sibling species of this complex. Our results show that intraspecific divergence is higher in *C. obsoletus* (*ss*) northern populations, and highlight the isolation of *C. obsoletus* (*ss*) populations in the Canary Islands. No cryptic species within *C. obsoletus* were identified.

## Supplementary Information

**Additional file 1**: **Table S1. **Spanish* cox1* georeferenced sequences retrieved from GenBank**.**

**Additional file 2**: **Table S2. **Pairwise distance of the haplotypes identified in this study based on a* p*-distance model.

## Data Availability

The generated sequences were submitted to the GenBank database under accession nos. MW602810-MW602832.

## Abbreviations

AHSV: African horse sickness virus
bp: Base pair
BTV: Bluetongue virus
cox1: Cytochrome *c* oxidase I
EHDV: Epizootic hemorrhagic disease virus
ML: Maximum likelihood
mtDNA: Mitochondrial DNA
PCR: Polymerase chain reaction
