# Supplementary material for: A study of the composition of the Obsoletus complex and genetic diversity of Culicoides obsoletus populations in Spain
Source: Parasit Vectors. 2021 Jul 3;14:351. doi: 10.1186/s13071-021-04841-z (PMC8254917; doi:10.1186/s13071-021-04841-z)
Supplement: Supplementary file 1 — Additional file 1: Table S1. Spanish cox1 georeferenced sequences retrieved from GenBank. [file 13071_2021_4841_MOESM1_ESM.docx]

**Table S1. Spanish cox1 georeferenced sequences retrieved from GenBank.**

| **Collection sites** | **ID^a^** | **Longitude** | **Latitude** | **Accession number** | **Observations** |
| --- | --- | --- | --- | --- | --- |
| Las Palmas de Gran Canaria | GC3 | -15.5 | 28.14 | JQ740596 | In the referenced paper (Martínez de la Puente et al, 2012) it is stated that 1 out of 11 sequenced midges was from this haplotype |
| Las Palmas de Gran Canaria | GC3 | -15.5 | 28.14 | JQ740594 | In the referenced paper (Martínez de la Puente et al, 2012) it is stated that 10 out of 11 sequenced midges were from this haplotype |
| Tenerife | TF | -16.37 | 28.52 | JQ740595 | In the referenced paper (Martínez de la Puente et al, 2012) it is stated that all 9 sequenced midges were from this haplotype |
| Torre del Campo | CO | -3.9 | 37.83 | MT171158 |  |
| Torre del Campo | CO | -3.9 | 37.83 | MT171159 |  |
| Torre del Campo | CO | -3.9 | 37.83 | MT171160 |  |
| Torre del Campo | CO | -3.9 | 37.83 | MT171161 |  |
| Torre del Campo | CO | -3.9 | 37.83 | MT171162 |  |
| Torre del Campo | CO | -3.9 | 37.83 | MT171163 |  |
| Torre del Campo | CO | -3.9 | 37.83 | MT171164 |  |
| Torre del Campo | CO | -3.9 | 37.83 | MT171165 |  |
| Torre del Campo | CO | -3.9 | 37.83 | MT171166 |  |
| Torre del Campo | CO | -3.9 | 37.83 | MT171167 |  |
| Torre del Campo | CO | -3.9 | 37.83 | MT171168 |  |
| Torre del Campo | CO | -3.9 | 37.83 | MT171169 |  |
| Torre del Campo | CO | -3.9 | 37.83 | MT171170 |  |
| Orgiva | AL | -2.99 | 36.85 | MT171171 |  |
| Orgiva | AL | -2.99 | 36.85 | MT171172 |  |
| Orgiva | AL | -2.99 | 36.85 | MT171173 |  |
| Orgiva | AL | -2.99 | 36.85 | MT171176 |  |
| Orgiva | AL | -2.99 | 36.85 | MT171177 |  |
| Orgiva | AL | -2.99 | 36.85 | MT171180 |  |
| Castril | GR | -2.34 | 37.73 | MT171181 |  |
| Castril | GR | -2.34 | 37.73 | MT171183 |  |
| Castril | GR | -2.34 | 37.73 | MT171184 |  |
| Castril | GR | -2.34 | 37.73 | MT171185 |  |
| Castril | GR | -2.34 | 37.73 | MT171186 |  |
| Castril | GR | -2.34 | 37.73 | MT171187 |  |
| Castril | GR | -2.34 | 37.73 | MT171188 |  |
| Castril | GR | -2.34 | 37.73 | MT171189 |  |
| Castril | GR | -2.34 | 37.73 | MT171190 |  |
| Castril | GR | -2.34 | 37.73 | MT171191 |  |
| Castril | GR | -2.34 | 37.73 | MT171192 |  |
| Castril | GR | -2.34 | 37.73 | MT171193 |  |
| Castril | GR | -2.34 | 37.73 | MT171194 |  |
| Castril | GR | -2.34 | 37.73 | MT171195 |  |
| Castril | GR | -2.34 | 37.73 | MT171196 |  |
| Castril | GR | -2.34 | 37.73 | MT171197 |  |
| Arauut | B | 1.76 | 41.97 | MT171200 |  |
| Arauut | B | 1.76 | 41.97 | MT171202 |  |
| Arauut | B | 1.76 | 41.97 | MT171203 |  |
| Arauut | B | 1.76 | 41.97 | MT171204 |  |
| Arauut | B | 1.76 | 41.97 | MT171205 |  |
| Arauut | B | 1.76 | 41.97 | MT171207 |  |
| Arauut | B | 1.76 | 41.97 | MT171208 |  |
| Arauut | B | 1.76 | 41.97 | MT171209 |  |
| Arauut | B | 1.76 | 41.97 | MT171210 |  |
| Arauut | B | 1.76 | 41.97 | MT171212 |  |
| Arauut | B | 1.76 | 41.97 | MT171213 |  |
| Arauut | B | 1.76 | 41.97 | MT171214 |  |
| Arauut | B | 1.76 | 41.97 | MT171215 |  |
| Arauut | B | 1.76 | 41.97 | MT171217 |  |
| Arauut | B | 1.76 | 41.97 | MT171218 |  |
| Arauut | B | 1.76 | 41.97 | MT171219 |  |
| Arauut | B | 1.76 | 41.97 | MT171220 |  |
| Arauut | B | 1.76 | 41.97 | MT171221 |  |
| Arauut | B | 1.76 | 41.97 | MT171222 |  |
| Arauut | B | 1.76 | 41.97 | MT171224 |  |
| Arauut | B | 1.76 | 41.97 | MT171225 |  |
| Arauut | B | 1.76 | 41.97 | MT171226 |  |
| Arauut | B | 1.76 | 41.97 | MT171227 |  |
| Arauut | B | 1.76 | 41.97 | MT171228 |  |
| Arauut | B | 1.76 | 41.97 | MT171229 |  |
| Arauut | B | 1.76 | 41.97 | MT171230 |  |
| Arauut | B | 1.76 | 41.97 | MT171231 |  |
| Arauut | B | 1.76 | 41.97 | MT171232 |  |
| Arauut | B | 1.76 | 41.97 | MT171233 |  |
| Arauut | B | 1.76 | 41.97 | MT171234 |  |
| Arucas | GC2 | -15.5 | 28.13 | MT171235 |  |
| Arucas | GC2 | -15.5 | 28.13 | MT171236 |  |
| Arucas | GC2 | -15.5 | 28.13 | MT171237 |  |
| Arucas | GC2 | -15.5 | 28.13 | MT171238 |  |
| Arucas | GC2 | -15.5 | 28.13 | MT171239 |  |
| Arucas | GC2 | -15.5 | 28.13 | MT171240 |  |
| Arucas | GC2 | -15.5 | 28.13 | MT171241 |  |
| Arucas | GC2 | -15.5 | 28.13 | MT171243 |  |
| Arucas | GC2 | -15.5 | 28.13 | MT171244 |  |
| Arucas | GC2 | -15.5 | 28.13 | MT171245 |  |
| Arucas | GC2 | -15.5 | 28.13 | MT171246 |  |
| Arucas | GC2 | -15.5 | 28.13 | MT171247 |  |
| Arucas | GC2 | -15.5 | 28.13 | MT171248 |  |
| Arucas | GC2 | -15.5 | 28.13 | MT171249 |  |
| Arucas | GC2 | -15.5 | 28.13 | MT171250 |  |
| Arucas | GC2 | -15.5 | 28.13 | MT171251 |  |
| Arucas | GC2 | -15.5 | 28.13 | MT171252 |  |
| Arucas | GC2 | -15.5 | 28.13 | MT171253 |  |
| Arucas | GC2 | -15.5 | 28.13 | MT171254 |  |
| Arucas | GC2 | -15.5 | 28.13 | MT171255 |  |
| Arucas | GC2 | -15.5 | 28.13 | MT171256 |  |
| Arucas | GC2 | -15.5 | 28.13 | MT171257 |  |
| Arucas | GC2 | -15.5 | 28.13 | MT171258 |  |
| Arucas | GC2 | -15.5 | 28.13 | MT171259 |  |
| Arucas | GC2 | -15.5 | 28.13 | MT171260 |  |
| Arucas | GC2 | -15.5 | 28.13 | MT171261 |  |
| Arucas | GC2 | -15.5 | 28.13 | MT171262 |  |
| Arucas | GC2 | -15.5 | 28.13 | MT171263 |  |
| Arucas | GC2 | -15.5 | 28.13 | MT171264 |  |
| Arucas | GC2 | -15.5 | 28.13 | MT171265 |  |
| Arucas | GC2 | -15.5 | 28.13 | MT171266 |  |
| Arucas | GC2 | -15.5 | 28.13 | MT171267 |  |
| Arucas | GC2 | -15.5 | 28.13 | MT171268 |  |
| Arucas | GC2 | -15.5 | 28.13 | MT171269 |  |
| Arucas | GC2 | -15.5 | 28.13 | MT171270 |  |
| Arucas | GC2 | -15.5 | 28.13 | MT171271 |  |
| Arucas | GC2 | -15.5 | 28.13 | MT171272 |  |
| Palma | IB | 2.65 | 39.56 | MT171273 |  |
| Palma | IB | 2.65 | 39.56 | MT171274 |  |
| Palma | IB | 2.65 | 39.56 | MT171275 |  |
| Palma | IB | 2.65 | 39.56 | MT171276 |  |
| Palma | IB | 2.65 | 39.56 | MT171277 |  |
| Palma | IB | 2.65 | 39.56 | MT171278 |  |
| Palma | IB | 2.65 | 39.56 | MT171279 |  |
| Palma | IB | 2.65 | 39.56 | MT171280 |  |
| Palma | IB | 2.65 | 39.56 | MT171281 |  |
| Palma | IB | 2.65 | 39.56 | MT171283 |  |
| Palma | IB | 2.65 | 39.56 | MT171284 |  |
| Palma | IB | 2.65 | 39.56 | MT171285 |  |
| Palma | IB | 2.65 | 39.56 | MT171286 |  |
| Palma | IB | 2.65 | 39.56 | MT171287 |  |
| Palma | IB | 2.65 | 39.56 | MT171288 |  |
| Palma | IB | 2.65 | 39.56 | MT171289 |  |
| Palma | IB | 2.65 | 39.56 | MT171290 |  |
| Palma | IB | 2.65 | 39.56 | MT171291 |  |
| Palma | IB | 2.65 | 39.56 | MT171292 |  |
| Palma | IB | 2.65 | 39.56 | MT171293 |  |
| Palma | IB | 2.65 | 39.56 | MT171294 |  |
| Palma | IB | 2.65 | 39.56 | MT171295 |  |
| Palma | IB | 2.65 | 39.56 | MT171296 |  |
| Palma | IB | 2.65 | 39.56 | MT171297 |  |
| Palma | IB | 2.65 | 39.56 | MT171298 |  |
| Palma | IB | 2.65 | 39.56 | MT171299 |  |

^a^the ID corresponds to the one assigned in this study
