# Supplementary material for: A study of the composition of the Obsoletus complex and genetic diversity of Culicoides obsoletus populations in Spain
Source: Parasit Vectors. 2021 Jul 3;14:351. doi: 10.1186/s13071-021-04841-z (PMC8254917; doi:10.1186/s13071-021-04841-z)
Supplement: Supplementary file 2 — Additional file 2: Table S2. Pairwise distance of the haplotypes identified in this study based on a p-distance model. [file 13071_2021_4841_MOESM2_ESM.docx]

**Table S2. Pairwise distance of the haplotypes identified in this study based on p-distance model.**

|  | **Ob01** | **Ob02** | **Ob03** | **Ob04** | **Ob05** | **Ob06** | **Ob08** | **Ob09** | **Ob07** | **Ob10** | **Ob11** | **Ob12** | **Ob13** | **Ob14** | **Ob15** | **Ob16** | **Ob17** | **Ob18** | **Ob19** | **Sc01** | **Sc02** | **Sc03** | **Mon01** |
| --- | --- | --- | --- | --- | --- | --- | --- | --- | --- | --- | --- | --- | --- | --- | --- | --- | --- | --- | --- | --- | --- | --- | --- |
| **Ob01** |  |  |  |  |  |  |  |  |  |  |  |  |  |  |  |  |  |  |  |  |  |  |  |
| **Ob02** | 0.0117 |  |  |  |  |  |  |  |  |  |  |  |  |  |  |  |  |  |  |  |  |  |  |
| **Ob03** | 0.0097 | 0.0019 |  |  |  |  |  |  |  |  |  |  |  |  |  |  |  |  |  |  |  |  |  |
| **Ob04** | 0.0117 | 0.0039 | 0.0019 |  |  |  |  |  |  |  |  |  |  |  |  |  |  |  |  |  |  |  |  |
| **Ob05** | 0.0058 | 0.0097 | 0.0078 | 0.0097 |  |  |  |  |  |  |  |  |  |  |  |  |  |  |  |  |  |  |  |
| **Ob06** | 0.0117 | 0.0078 | 0.0058 | 0.0078 | 0.0058 |  |  |  |  |  |  |  |  |  |  |  |  |  |  |  |  |  |  |
| **Ob08** | 0.0156 | 0.0097 | 0.0078 | 0.0058 | 0.0097 | 0.0078 |  |  |  |  |  |  |  |  |  |  |  |  |  |  |  |  |  |
| **Ob09** | 0.0117 | 0.0078 | 0.0058 | 0.0078 | 0.0058 | 0.0039 | 0.0078 |  |  |  |  |  |  |  |  |  |  |  |  |  |  |  |  |
| **Ob07** | 0.0136 | 0.0058 | 0.0039 | 0.0058 | 0.0078 | 0.0058 | 0.0078 | 0.0058 |  |  |  |  |  |  |  |  |  |  |  |  |  |  |  |
| **Ob10** | 0.0117 | 0.0039 | 0.0019 | 0.0039 | 0.0058 | 0.0039 | 0.0058 | 0.0039 | 0.0019 |  |  |  |  |  |  |  |  |  |  |  |  |  |  |
| **Ob11** | 0.0117 | 0.0058 | 0.0039 | 0.0058 | 0.0058 | 0.0039 | 0.0039 | 0.0039 | 0.0039 | 0.0019 |  |  |  |  |  |  |  |  |  |  |  |  |  |
| **Ob12** | 0.0097 | 0.0058 | 0.0039 | 0.0058 | 0.0039 | 0.0019 | 0.0058 | 0.0019 | 0.0039 | 0.0019 | 0.0019 |  |  |  |  |  |  |  |  |  |  |  |  |
| **Ob13** | 0.0136 | 0.0078 | 0.0058 | 0.0039 | 0.0078 | 0.0058 | 0.0019 | 0.0058 | 0.0058 | 0.0039 | 0.0019 | 0.0039 |  |  |  |  |  |  |  |  |  |  |  |
| **Ob14** | 0.0156 | 0.0117 | 0.0097 | 0.0078 | 0.0097 | 0.0039 | 0.0078 | 0.0078 | 0.0097 | 0.0078 | 0.0078 | 0.0058 | 0.0058 |  |  |  |  |  |  |  |  |  |  |
| **Ob15** | 0.0136 | 0.0097 | 0.0078 | 0.0097 | 0.0078 | 0.0058 | 0.0097 | 0.0019 | 0.0078 | 0.0058 | 0.0058 | 0.0039 | 0.0078 | 0.0097 |  |  |  |  |  |  |  |  |  |
| **Ob16** | 0.0136 | 0.0058 | 0.0039 | 0.0058 | 0.0117 | 0.0097 | 0.0117 | 0.0097 | 0.0078 | 0.0058 | 0.0078 | 0.0078 | 0.0097 | 0.0136 | 0.0117 |  |  |  |  |  |  |  |  |
| **Ob17** | 0.0097 | 0.0136 | 0.0117 | 0.0136 | 0.0039 | 0.0097 | 0.0136 | 0.0097 | 0.0117 | 0.0097 | 0.0097 | 0.0078 | 0.0117 | 0.0136 | 0.0117 | 0.0117 |  |  |  |  |  |  |  |
| **Ob18** | 0.0156 | 0.0117 | 0.0097 | 0.0117 | 0.0097 | 0.0078 | 0.0117 | 0.0039 | 0.0097 | 0.0078 | 0.0078 | 0.0058 | 0.0097 | 0.0117 | 0.0058 | 0.0136 | 0.0136 |  |  |  |  |  |  |
| **Ob19** | 0.0156 | 0.0097 | 0.0078 | 0.0058 | 0.0097 | 0.0078 | 0.0039 | 0.0078 | 0.0078 | 0.0058 | 0.0039 | 0.0058 | 0.0019 | 0.0078 | 0.0097 | 0.0117 | 0.0136 | 0.0117 |  |  |  |  |  |
| **Sc01** | 0.1245 | 0.1206 | 0.1187 | 0.1167 | 0.1226 | 0.1206 | 0.1187 | 0.1187 | 0.1167 | 0.1187 | 0.1187 | 0.1187 | 0.1167 | 0.1167 | 0.1206 | 0.1187 | 0.1206 | 0.1187 | 0.1187 |  |  |  |  |
| **Sc02** | 0.1226 | 0.1187 | 0.1167 | 0.1148 | 0.1206 | 0.1187 | 0.1167 | 0.1167 | 0.1148 | 0.1167 | 0.1167 | 0.1167 | 0.1148 | 0.1148 | 0.1187 | 0.1167 | 0.1187 | 0.1167 | 0.1167 | 0.0039 |  |  |  |
| **Sc03** | 0.1226 | 0.1187 | 0.1167 | 0.1148 | 0.1206 | 0.1187 | 0.1167 | 0.1167 | 0.1148 | 0.1167 | 0.1167 | 0.1167 | 0.1148 | 0.1148 | 0.1187 | 0.1167 | 0.1187 | 0.1167 | 0.1167 | 0.0019 | 0.0019 |  |  |
| **Mon01** | 0.0331 | 0.0311 | 0.0331 | 0.0350 | 0.0292 | 0.0311 | 0.0350 | 0.0311 | 0.0331 | 0.0311 | 0.0311 | 0.0292 | 0.0331 | 0.0350 | 0.0331 | 0.0331 | 0.0292 | 0.0350 | 0.0350 | 0.1304 | 0.1284 | 0.1284 |  |
